# Rethinking Emergent Literacy in Children With Hearing Loss

**DOI:** 10.3389/fpsyg.2020.00039

**Published:** 2020-01-31

**Authors:** Erin M. Ingvalson, Tina M. Grieco-Calub, Lynn K. Perry, Mark VanDam

**Affiliations:** ^1^School of Communication Science and Disorders, Florida State University, Tallahassee, FL, United States; ^2^Roxelyn and Richard Pepper Department of Communication Sciences and Disorders, Northwestern University, Evanston, IL, United States; ^3^Department of Psychology, University of Miami, Coral Gables, FL, United States; ^4^Department of Speech and Hearing Sciences, Washington State University, Spokane, WA, United States

**Keywords:** emergent literacy, hearing loss, phonological awareness, vocabulary development, language development, cochlear implants, morphosyntax

## Abstract

Long-term literacy outcomes for children with hearing loss, particularly those with severe-to-profound deafness who are fitted with cochlear implants (CIs) lag behind those of children with normal hearing (NH). The causes for these long-term deficits are not fully clear, though differences in auditory access between children who use CIs and those with NH may be a partial cause. This paper briefly reviews the emergent literacy model as proposed by Whitehurst and Lonigan (1998). We then examine the development of each of Whitehurst and Lonigan’s identified factors in children who use CIs and how the extant knowledge of language and literacy development in children who use CIs may bear on the emergent literacy model. We then propose to modify the model for children who use CIs based on their unique developmental trajectories, influenced at least in part by their unique auditory access. We conclude with future directions for further development of an evidence-based emergent literacy model for children who use CIs and how this model could be used to inform intervention.

## Introduction

Reading is a critical skill that is typically acquired in childhood. Reading skill, acquired knowledge, and academic success increase together (Whitehurst and Lonigan, 2001). Cultivated reading skills advance the ability to read, enhance language skills, and beget cascading success in other domains dependent on reading for knowledge (Kutner, 2007; Newman, 2009). Unsurprisingly, reading deficits or delays in young children hamper success across academic domains. This leads to a rich-get-richer and poor-get-poorer effect (Whitehurst and Lonigan, 1998), in which the best readers find success and the poorer readers are at risk of falling progressively behind.

Cochlear implants have provided access to sound to thousands of prelingually deafened children. Along with this access to sound, many of these children have experienced substantial gains in their spoken language skills (Tomblin et al., 2005; Geers et al., 2008; Niparko et al., 2010). Despite these substantial gains, children who use CIs continue to lag behind their peers with normal hearing (NH) on measures of spoken language. This is also true on measures of literacy. Measured as a whole, children with varying degrees of hearing loss, regardless of whether they use amplification or not or the type of communication mode that they use (e.g., spoken language or manual communication), score below their NH peers on literacy performance at high school graduation (Scarborough, 2001; Qi and Mitchell, 2012). Looking specifically at the subset of children who use CIs, fewer than half (44%) of children are reading at grade level when graduating high school (Geers et al., 2008).

A major factor contributing to the poor high school reading level by children with CIs is that they tend to score significantly below their peers with NH on *emergent* literacy factors at kindergarten (Easterbrooks et al., 2008; Nittrouer et al., 2012). This means that children who use CIs begin formal schooling with literacy skills that are already behind their peers with NH (Scarborough, 2001). Given the long-term deficits in literacy performance for children who use CIs and are spoken language users, interventions that target emergent literacy skills could provide a foundation on which future literacy development is built. Our aim here is to provide an overview of emergent literacy in children who use CIs, reviewing how the pre-kindergarten skills of these children compare to their peers with NH.

Before we can consider emergent literacy development in children who use CIs, however, we need to consider the unique auditory experience in children who use CIs compared to that of their typically developing peers with NH during early childhood. Within the first year of life, children with NH show refined acoustic frequency and intensity discrimination (as reviewed in Werner et al., 2012). With additional experience, children develop a heightened perceptual sensitivity to phonetic contrasts within their native language and a loss of perceptual sensitivity to non-native phonetic contrasts (Werker and Tees, 1984). In addition, as infants become more proficient at tracking statistical probabilities within their world, they are able to discriminate speech sounds that co-occur frequently from those that do not (Saffran et al., 1996), which appears to prepare them for building receptive vocabulary (Graf Estes et al., 2007). Within the second year of life, children with NH rapidly acquire vocabulary words and become increasingly proficient at processing speech in real time (Fernald et al., 1998). Thus, children with normal hearing are quite proficient at processing the speech in their language by the end of the second year of life, and this proficiency has positive cascading effects for future language and cognitive development (Marchman and Fernald, 2008).

The auditory experience of children who use CIs, however, is quite different. For example, most children who use CIs experience complete auditory and language deprivation, either at or shortly after birth, prior to the activation of their CIs. Even after CI activation, children’s access to speech may be highly variable. This variability arises from both technical and biological variability of CIs and their interface with the children’s auditory systems. Specifically, the CI can have poor surgical insertion, resulting in altered placement of the electrode array (Cakir et al., 2016). In addition, there may be channel interaction, whereby different electrodes are stimulating different populations of neurons. Or, there may be biological variability in how the children’s auditory system transmits the electrical signal provided by the CI (DeVries et al., 2016). As a result, there is often variability in children’s recognition of speech even after early and intensive auditory-only training (Geers et al., 2009; Davidson et al., 2011).

Poor recognition of speech places children at risk for delayed spoken language development (Davidson et al., 2011; Geers and Hayes, 2011). Delayed spoken language development in turn places children at risk for delayed literacy development (Easterbrooks et al., 2008; Webb et al., 2015). The inability to distinguish individual speech units, such as phonemes that comprise words, due to poor frequency resolution, may disrupt the development of phonological categories and limit children’s ability to use partial phonological knowledge to process speech incrementally (Nittrouer et al., 2014). Disrupted phonological categories and poor speech processing can impact a child’s ability to develop phonological awareness (James et al., 2005, 2009). Clearly, then, children who use CIs face risks to their literacy development across multiple fronts, beginning almost at birth.

## Literacy Model for Typically Developing Children

The emergent literacy model says that literacy is an incrementally acquired skill that includes literacy-related behaviors and complex, interdependent developmental relationships among factors such as pre-reading behaviors and the environments that influence social skills (Reese et al., 2010). Consistent with this model, Whitehurst and Lonigan posit an intersection of a child’s ability to decode print units into sound units and then place these sound units into a meaningful context. In their original conceptualization, they suggested these skills could be best described as “outside-in” and “inside-out” processes of emergent literacy (Whitehurst and Lonigan, 1998, 2001). Outside-in processes are what and how children understand from reading, including semantic knowledge and narrative; the skills come from outside the printed word. Inside-out processes are how children understand print as sounds and words, including alphabetic knowledge, phonological awareness, and print awareness; the skills come from inside the printed word. Language units, such as vocabulary knowledge and morphosyntax, represent the intersection of outside-in and inside-out processes. Ongoing research has repeatedly demonstrated that these processes are critical components of emergent literacy to allow children to successfully decode sentences and to extract the meaning of the sentence and place it in context (Sénéchal et al., 2001), but they are now more often divided into print awareness, phonological awareness, and oral language skills. Added to these processes are also influences of cognitive ability, interest, attention, and external factors including cultural norms and SES (Dickinson and Tabors, 2001; Sirin, 2005).

It has also been suggested that print awareness and phonological awareness skills are important in earlier stages of emergent reading and phonological awareness and oral language skills may play a greater role as complexity and reading skill increases later in development (Justice, 2006). This suggests that a structured approach to investigate the locus of domain-specific skills may be warranted. In particular, investigating the co-development of print and phonological awareness skills in early stages may provide clearer information regarding the locus of literacy deficits for children who use CIs than is currently available. In the following sections, we summarize the research into specific skills related to emergent literacy for children who use CIs. We focus our review primarily on the preschool period, when children are developing the skills that will support learning to read once they enter kindergarten. As such, our review will focus primarily on children’s development of skills that support decoding, including phonological awareness and vocabulary development. Our emphasis is on emergent literacy because, as noted above, children who use CIs show deficits in emergent literacy skills at the start of kindergarten and deficits in literacy performance persist throughout their academic careers. By considering the development of literacy in this early period, we may be able to create interventions that give children a foundation for literacy development.

## Print Awareness

The work to-date into development of alphabetic knowledge and print awareness by preschoolers who use CIs generally indicates that this is an area of strength. When comparing preschoolers who use CIs and preschoolers with NH on alphabetic knowledge, Ambrose et al. found that the two groups were not statistically different (Ambrose et al., 2012). Easterbrooks et al. took a slightly different approach, assessing alphabetic knowledge from a variety of preschoolers with hearing loss who used amplification (both CIs and hearing aids) without assessing a parallel group of children with NH. They reported that the performance on alphabetic knowledge for children with hearing loss fell within the standardized norms for children with NH (Easterbrooks et al., 2008). Though they did not report the data for children who use CIs separately from those who used hearing aids, their finding that alphabetic knowledge is an area of strength is consistent with extant research. Finally, children in the Longitudinal Outcomes of Children with Hearing Impairment (LOCHI) cohort had word attack and word identification skills within NH norms at age 5 (Ching et al., 2014).

A recent study by Werfel et al. (2015) expands on our knowledge of print awareness in children with hearing loss, including both CI users and hearing aid users. They observed that the literature to date had focused on children’s alphabetic knowledge, but that print awareness is a broader concept that includes print concept knowledge and word concept knowledge (e.g., Justice and Ezell, 2001; Levy et al., 2006). Their sample included eight preschoolers with severe-to-profound hearing loss who used either CIs and/or hearing aids and all of whom were exclusively spoken language users. They replicated earlier findings that preschoolers with hearing loss were not significantly different from children with NH on measures of alphabetic knowledge, but preschoolers with hearing loss showed deficits relative to preschoolers with NH on measures of print and word concept knowledge. They suggested this disparity might be due to differences in shared storybook reading practices between caregivers and children with hearing loss relative to caregivers and children with NH but note that this conclusion is preliminary and requires further investigation into practices such as shared joint attention by children with hearing loss.

## Phonological Awareness

Phonological awareness has long been identified as one of the most predictive skills of young children’s future decoding abilities (Wagner et al., 1997). Given this importance, it has received a fair amount of attention in the literature (e.g., Stahl and Murray, 1994; Swank and Catts, 1994; Bus and van IJzendoorn, 1999; Carroll et al., 2003; Foy and Mann, 2003; Mann and Foy, 2007; Phillips et al., 2008; Bridges and Catts, 2011; Hulme et al., 2012). This work has demonstrated that the broad concept of phonological awareness can be broken down into phonological sensitivity, phonological memory, and phonological naming, all of which develop during the preschool period (Whitehurst and Lonigan, 2001). We review the performance of preschoolers who use CIs on each sub-skill below.

### Phonological Sensitivity

Phonological sensitivity refers to the ability to isolate, segment, manipulate, or identify in some other way the sound components of language (Stanovich, 1992). In preschoolers, this is often assessed *via* rhyming, blending, and elision tasks at the word, syllable, and/or phoneme level (Anthony et al., 2002; Anthony and Lonigan, 2004). These studies have demonstrated that phonological sensitivity may be a unidimensional construct that progresses from the sensitivity to phonological units at the word level to sensitivity at higher levels of complexity such as the individual phoneme (Wagner et al., 1994, 1997).

Overall, children who use CIs have the greatest deficits relative to children with NH at segment-level tasks, with relatively strong performance on word-level and syllable-level tasks (James et al., 2005). Focusing on the preschool period, preschoolers who use CIs are poorer than preschoolers with NH at sound blending (Ambrose et al., 2012). Even 4 years post-CI activation, children who use CIs continue to perform significantly below NH norms on the ability to segment speech into sound components (Spencer and Tomblin, 2008). Rhyme is another area of persistent weakness (Easterbrooks et al., 2008; James et al., 2009). Assessed in the fall of a given school year, 67% of preschoolers with hearing loss were unable to complete a rhyme task (the sample included both CI and hearing aid users; Easterbrooks et al., 2008). When these same children were retested in the following spring, 45% of them remained unable to complete the rhyming task.

### Phonological Memory

The phonological memory skills of children who use CIs have been of great scientific interest in recent years, and some consider it one of the core deficits in this population (Pisoni et al., 2016). School-aged children who use CIs are significantly poorer than aged-matched peers with NH on measures of phonological memory including digit span (Dawson et al., 2002; Burkholder and Pisoni, 2003; Pisoni and Cleary, 2003), nonword repetition (Carter et al., 2002; Watson et al., 2007), and sequencing skills (Cleary et al., 2001; Conway et al., 2010). Though the phonological memory performance of children with CIs is often measured when they are in elementary school, their performance deficits are hypothesized to begin much earlier, resulting from a lack of auditory input in the pre-implant period (Conway et al., 2009; though see Hall et al., 2017, for an alternative hypothesis). Preschoolers who use CIs do have performance deficits in verbal working memory overall (Beer et al., 2011, 2014), suggesting that they may indeed have deficits in phonological memory in particular.

### Phonological Naming

Phonological naming, sometimes referred to as rapid automatic naming, refers to the child’s ability to efficiently retrieve the phonological codes from long-term storage in order to name a series of pictures, colors, or digits. This skill is thought to be related to a child’s ability to efficiently access stored phonological information necessary for decoding (Norton and Wolf, 2012). Like alphabetic knowledge, phonological naming appears to be an area of strength for children who use CIs (Sahlén et al., 2004; Reuterskiöld et al., 2010; Werfel, 2017). As a note of caution, however, these studies are often conducted on school-aged children who are a mix of CI and hearing aid users. Thus, we cannot be certain that preschoolers who use CIs have age-typical phonological naming performance. One exception is Werfel (2017), who measured preschoolers with hearing loss (both CI and hearing aid users) and found that preschoolers with hearing loss were not significantly different from children with NH on phonological naming. With that caveat, age-typical performance on phonological naming would be consistent with children’s relative strengths on phonological sensitivity tasks that assess their abilities at the word or syllable level. This in turn is in line with their difficulties recognizing speech resulting from poor frequency resolution in the CI. In summary, children who use CIs are likely to develop poor emergent literacy skills in those domains that depend on strong speech segmentation but may be able to develop age-typical emergent literacy skills in domains where poor frequency resolution is less likely to impact skill development. In the subsequent sections, we will consider how poor speech segmentation following implantation impacts language development, and how this influences emergent literacy.

## Oral Language

Oral language is a broad skillset, encompassing skills such as vocabulary and morphosyntax as well as higher-level language skills such as semantic knowledge and narrative abilities necessary for placing information in context. As children progress in their reading ability and shift from primarily decoding, oral language skills can play a larger role in supporting literacy comprehension (this progression is described by the Simple View of Reading; Gough and Tunmer, 1986). But even in the preschool period, oral language skills play a role in emergent literacy development, where relationships between vocabulary and phonological sensitivity are well documented (Metsala, 1999; Walley et al., 2003).

### Vocabulary

Although children who use CIs are a heterogeneous group, they often lag behind their peers with NH in vocabulary development (Nott et al., 2009; Ganek et al., 2012; Lund, 2016). Evidence for these lags in vocabulary development comes from both standardized assessments and novel word learning tasks in which the child’s ability to retain a novel word-referent association and/or generalize those novel words to new instances of a category are tested.

Several studies using standardized assessments find children who use CIs who are implanted early have vocabularies within the NH range by school entry (Connor et al., 2000; Geers et al., 2009). However, most studies, especially those that directly compare the vocabularies of children who use CIs to those who do not rather than referencing norms, show that children who use CIs have smaller vocabularies than age-matched peers (El-Hakim et al., 2001; Nott et al., 2009; Davidson et al., 2014b; and see Lund, 2016 for a meta-analysis). Critically, both types of studies tend to find that earlier implantation is associated with larger vocabularies and better outcomes (with the caveat that earlier implantation is often confounded with longer duration of use, Dunn et al., 2014; but see Nicholas and Geers, 2018, which suggests that age of implantation may provide a unique benefit).

Outside of vocabulary size, studies of semantic knowledge look to understand children’s conceptual knowledge and their ability to learn new words (Moeller et al., 2007). Despite the wealth of studies measuring vocabulary with standardized assessment, there is an overall lack of research on the processes by which children who use CIs learn new words. The studies that have focused on process tend to find that children who use CIs show comparable learning abilities with vocabulary-matched rather than age-matched peers at each stage of the word-learning processes. In particular, children who use CIs learn fewer novel words over the course of an experimental session than their age-matched peers with NH (Lederberg et al., 2000; Tomblin et al., 2007; Walker and McGregor, 2013). Such rapid word learning seems dependent on early auditory experience as age of implantation is correlated with the in-the-moment word-learning abilities of children who use CIs (Houston et al., 2012). Children’s access to sound during the learning period also matters, as children who use CIs with relatively good audibility perform better in novel word learning tasks than their peers with poor audibility, although still not at the levels of children with NH (Davidson et al., 2014b). This rapid word learning also has cascading consequences for children’s long-term outcomes (Connor et al., 2006), highlighting the important role that early vocabulary acquisition plays in children’s subsequent development.

In addition to mapping a word to a referent and remembering that mapping, successful word learning requires being able to generalize words to new instances of a category. Children with NH acquire a bias to attend to object shape, rather than color or material, helping them remember and generalize words (Perry et al., 2010; Perry and Samuelson, 2011). This bias in visual attention comes from regularity in visual experiences: most early learned words name categories of objects similar in shape (e.g., ball and car). As a group, however, preschool children who use CIs have difficulty compared to their peers in remembering the shapes of newly learned objects (Quittner et al., 2016) and accurately using relevant visual information to generalize words to new objects (Walker and McGregor, 2013). Children who use CIs also show delays in their ability to categorize familiar objects at the superordinate level, performing more similarly to vocabulary-matched than age-matched peers (Lund and Dinsmoor, 2016).

Together these data suggest that children who use CIs exhibit delays in their ability to map a new word to a referent, remember that association, generalize it to new category instances, and think about it flexibly as a member of a hierarchical category structure—the skills needed to acquire vocabulary. Overall, the research suggests that children who use CIs behave more similarly to vocabulary-matched than age-matched children, highlighting that the act of learning new words makes a child a better word learner, teaching them how to learn words (c.f., Smith et al., 2002). In this way, vocabulary acquisition sets the stage for subsequent language and literacy development.

### Morphosyntax

Deficits in morphosyntax are generally linked to the overall expressive language deficits observed in children who use CIs (Svirsky et al., 2000). For example, kindergarteners who use CIs have significantly shorter utterances than kindergarteners with NH. Looking at particular language structures, the children who use CIs produce significantly fewer bound morphemes, conjunctions, personal pronouns, and unique words (Nittrouer et al., 2014). Perhaps not surprisingly, then, only one-third of preschoolers who use CIs are producing utterance lengths and using bound morphemes at age-typical rates (Geers et al., 2016). A recent study compared preschoolers with hearing loss, including both CI and hearing aid users, to age- and language-matched groups of children with NH (Werfel, 2018). The study results showed that children with hearing loss had significantly more production errors on bound morphemes than children with NH, but the two groups were not significantly different on production of free morphemes; within tense, children with hearing loss were significantly less accurate on regular past-tense morphemes but were as accurate as children with NH on irregular third-person singular. Based on these data, Werfel concluded the morphosyntax delays stemmed at least in part from children’s perceptual deficits. Nicholas and Geers (2018) also found that perceptual access at least partially accounts for the morphosyntax abilities of preschoolers who use CIs, but with the caveat that age of access matters. They collected utterance lengths and numbers of bound morphemes produced from preschoolers who use CIs and preschoolers with NH. Early implanted preschoolers (before 11 months) were not significantly different from preschoolers with NH on the morphosyntax measures. But preschoolers who were implanted later, even after equating duration of CI use, did not receive the same benefit. These later-implanted children showed growth in expressive language, including morphosyntax, during preschool, but continued to show significant performance deficits relative to children with NH.

### Narrative

Narrative abilities tie closely to morphosyntax abilities in that children’s expressive language is closely tied to their ability to produce a coherent narrative (Bamberg and Damrad-Frye, 1991). Not surprisingly then, children who use CIs have deficits in their narrative abilities relative to children with NH (Crosson and Geers, 2001). Again, perhaps not surprisingly, children who use CIs vary greatly in their narrative performance: those children whose speech perception scores fall in the normal range had narratives that included the same components as children with NH whereas those whose speech perception scores were lower did not (Crosson and Geers, 2001). Children who use CIs show particular deficits in narrative micro-structure, which includes elements such as tense marking and referents (Jones et al., 2016). Children’s narrative micro-structure was best predicted by their receptive vocabulary scores. Thus, it seems that weaknesses in narrative micro-structure are closely tied to existent deficits found in children with CIs in vocabulary development and expressive morphosyntax. Though it remains to be empirically verified, this could suggest that improvements in vocabulary and morphosyntax could lead to more cohesive narratives.

## Rethinking Emergent Literacy in Children who use Cochlear Implants

In the preceding sections, we have summarized areas of strength and weakness in emergent literacy for children who use CIs. We see that children’s performance is age-typical on those measures that are not impacted by their auditory access, including alphabetic knowledge and phonological naming. In contrast, children who use CIs are significantly poorer than age-matched peers with NH on measures of phonological sensitivity, phonological memory, and most measures of oral language, all of which rely on good speech segmentation. It appears, then, that children who use CIs have trouble relative to their peers with NH in mastering the skills important for decoding, which will then lead to downstream trouble in success with comprehension.

To facilitate intervention efforts in improving emergent literacy, and thereby decoding skills, in children who use CIs, we suggest rethinking the emergent literacy model for this population. We take as our starting point the three components of emergent literacy theorized by Whitehurst and Lonigan. We choose this model as our starting point for two reasons. First, the model originally put forth by Whitehurst and Lonigan described the components important for typically developing prereaders, with the presumption that though these components may be distinguishable, they are interdependent. Work building off this model has reinforced the importance of not only these individual components but also how they interact during development to promote emergent literacy during the preschool period (Anthony et al., 2002; Anthony and Lonigan, 2004; Lerner and Lonigan, 2016). By taking the components and developmental trajectories of emergent literacy as our baseline, we aim to begin considering a model of emergent literacy for prereaders who use CIs, considering those areas where they differ from typical development as potential targets for intervention. Second, though the original 1998 model split skills into “outside-in” and “inside-out,” more modern conceptualizations describe a three-way split into print awareness, phonological awareness, and oral language (the convention followed here). We believe that this three-factor model may be of particular benefit for children who use CIs, because it factors out print awareness—a strength for children who use CIs—from phonological awareness and oral language—skills that are generally not age-typical. Importantly, we believe the purpose of a model of emergent literacy for prereaders who use CIs is not simply identifying the components of emergent literacy, but theorizing how these components may influence one another during development (e.g., Scarborough, 2001), which has been crucial for driving empirical work and creating effective literacy interventions for children with literacy disorders (Snowling and Hulme, 2012). Our model therefore emphasizes interactions among the identified components. We fully anticipate that these hypotheses will be refined following empirical work, and we look forward to these efforts, as they will further our efforts to improve literacy outcomes for children who use CIs.

**Figure 1:**
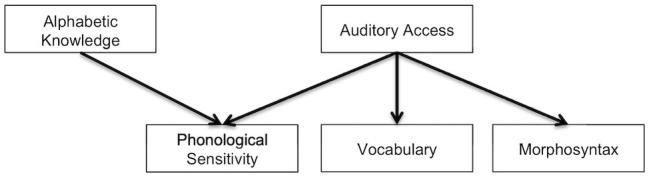
Schematic of our emergent literacy model for prereaders who use cochlear implants. We hypothesize that development on phonological sensitivity, vocabulary, and morphosyntax are correlated but that development on each construct does not influence growth on another. Rather, development on all three constructs is influenced primarily by children’s auditory access, particularly their ability to segment speech. Additionally, alphabetic knowledge is an area of strength for children who use cochlear implants, and we suspect that children’s phonological sensitivity may also build on their alphabetic knowledge. The unidirectional relationships described here are in contrast to bi-directional relationships between alphabetic knowledge and phonological sensitivity, vocabulary and morphosyntax, and vocabulary and phonological sensitivity that have been hypothesized for children with normal hearing.

As a final note before we present the model, a schematic of which is in Figure 1, we wish to re-emphasize that our focus is on prereaders who use CIs. Thus, the interaction among components described below is not likely to be identical in children who are native signers, nor is it likely to follow the same trajectory in children who have mild-to-moderate hearing loss and who use hearing aids (for a broader overview of early literacy including these populations see Easterbrooks et al., 2008). Also, because the emphasis is on emergent literacy, we are focusing primarily on preschool-aged children’s development of those skills that will support decoding. Our goal is to better conceptualize and treat decoding skills in the prereading years in order to better support efficient reading and comprehension in later elementary school (e.g., Gough and Tunmer, 1986; Scarborough, 2001).

### Co-development of Print Awareness and Phonological Sensitivity

The first arm of our new model is the co-development of alphabetic knowledge and phonological sensitivity. We focus solely on the phonological sensitivity arm of phonological awareness because this skill has been shown to be predictive of children’s future decoding abilities (Wagner et al., 1994, 1997), it has been the focus of much attention in the prereading abilities of children with NH (Anthony et al., 2002; Anthony and Lonigan, 2004), and it is an area of persistent weakness for children who use CIs (Easterbrooks et al., 2008; Ambrose et al., 2012; Werfel, 2017). In preschool children with NH, these two factors have a bidirectional relationship: growth on phonological sensitivity influences development in alphabetic knowledge and vice versa (Burgess and Lonigan, 1998; Lerner and Lonigan, 2016). Because alphabetic knowledge is an area of strength in children who use CIs (Ambrose et al., 2012; Werfel, 2017), we suggest that a different pattern may occur for these children. Children who use CIs may be able to use their relatively strong alphabetic knowledge skills as a foundation on which to build their emerging phonological sensitivity. In this scenario, growth in alphabetic knowledge would influence development in phonological sensitivity, but no influence would be seen from phonological sensitivity to alphabetic knowledge.

In a confirmatory factor analysis of emergent literacy in preschoolers with hearing loss, Webb et al. (2015) found that letter sounds loaded onto both the alphabetic knowledge and phonological awareness constructs. Letter sounds are often considered a print awareness measure (Ambrose et al., 2012; Webb et al., 2015), and its loading onto both factors could indicate that children are using the visual information available in print to help them learn sound segments (Easterbrooks et al., 2008; Dillon et al., 2012). Consistent with this hypothesis, in school-age readers who use CIs, those programs that use print materials as part of the phonological awareness training—such as by teaching sound-letter correspondence—show growth on general phonological awareness skills (Lederberg et al., 2014), providing evidence that an approach that builds on children’s existing strengths may lead to greater literacy success. We do not claim that auditory access plays no role in phonological sensitivity development for preschoolers who use CIs, rather, we argue that print information can help children resolve their poor spectral resolution to learn speech segments.

Alternatively, it is possible that phonological sensitivity performance by children who use CIs is primarily an indicator of their auditory ability (Davidson et al., 2014a). In this event, it would disconfirm our hypothesis that alphabetic knowledge is a leading indicator on phonological sensitivity for prereaders who use CIs; rather, it may be that the two skills develop somewhat independently with alphabetic knowledge developing following the timecourse observed in children with NH and phonological sensitivity being dependent on the ability of children who use CIs to segment speech. If phonological sensitivity is found to develop independently from alphabetic knowledge, then this would suggest a continuation of intervention approaches that emphasize phonological sensitivity training, often in an auditory-only manner to encourage successful sound manipulation (Lederberg et al., 2013; Werfel and Schuele, 2014). However, we note that though these interventions demonstrate gains on trained sounds, there is limited evidence of general skill acquisition (Werfel and Schuele, 2014), whereas children trained on intervention approaches that include alphabetic knowledge show more evidence acquiring general phonological sensitivity skill (Lederberg et al., 2014). We therefore suggest that, even if our model hypothesis is disconfirmed and alphabetic knowledge is not a leading indicator on phonological sensitivity, interventions that include alphabetic knowledge as a foundation for phonological sensitivity learning will be beneficial for prereaders who use CIs.

The reader will note that we have emphasized the relationship between alphabetic knowledge and phonological sensitivity, omitting the print concept knowledge portion of print awareness. We argue that phonological sensitivity may build on alphabetic knowledge in particular due first to the relationship between sound symbols and sound knowledge, and second because print concepts may not be as strong as alphabetic knowledge for children with HL (Werfel et al., 2015). Print concepts may benefit from increases in shared book reading between children with HL and their caregivers (DesJardin et al., 2008), and we anticipate that increases in exposure to print concepts could remediate this deficit.

### Co-development of Oral Language and Phonological Sensitivity

Because children who use CIs lag behind children with NH on measures of spoken language development (e.g., Geers et al., 2008, 2009), one question is whether phonological sensitivity performance deficits in children who use CIs stem from their general language performance deficits. There is some evidence suggesting this may be the case. Receptive vocabulary and phonological sensitivity are correlated for school-aged children who use CIs (James et al., 2008; Ettlinger and Johnson, 2010; Dillon et al., 2012), as they are for school-aged children with NH. In prereaders with NH, vocabulary is thought to support phonological sensitivity because children require more fine-grained phonological representations to differentiate words when they have more lexical competitors (Metsala, 1999; Werker and Stager, 2000; Walley et al., 2003). This would mean that improving vocabulary skills of children who use CIs could lead to gains in the children’s phonological sensitivity (Lund, 2016). In this scenario, interventions that target children’s word-learning abilities—noting that children who use CIs show deficits not only in overall vocabulary but in their word-learning abilities—would promote vocabulary development and phonological sensitivity growth.

A second possibility is that vocabulary development does not influence phonological sensitivity during emergent literacy development. Closer examination of the vocabulary-phonological awareness relationship in children with NH suggests that vocabulary growth may not impact phonological awareness development (Burgess and Lonigan, 1998; Lerner and Lonigan, 2016), and it is possible that the same mechanism may be at play in children who use CIs. In this scenario, poor performance in both domains by children who use CIs would stem from their overall language deficits (Castles and Coltheart, 2004), or perhaps more precisely, from their speech segmentation abilities. Because both phonological sensitivity and vocabulary knowledge in children who use CIs can be attributed to their auditory access, we hypothesize an uncoupled relationship may best describe the relationship between vocabulary and phonological sensitivity for children who use CIs. We would explain the apparent relationship between vocabulary and phonological sensitivity as recognizing that though vocabulary does not directly influence phonological sensitivity, the two constructs remain correlated (Burgess and Lonigan, 1998; Lerner and Lonigan, 2016): as children learn to use their CI, their speech segmentation skills improve, and along with that both their vocabulary and phonological sensitivity skills improve. To this end, though improving vocabulary knowledge for children who use CIs is necessary for future literacy success (Lund, 2016), doing so may not lead to phonological awareness gains. It is possible that vocabulary could mediate the relationship between auditory access and phonological sensitivity (as it appears to do for children who are hearing aid users, see Walker et al., 2019), which would also explain the correlations between vocabulary and phonological sensitivity found in school-aged children who use CIs. We suggest that in the event that vocabulary does serve as a mediator, it does so after children have passed through the prereading stage, when they have learned more words.

Another important component of developing language is morphosyntax. Relative to the vocabulary-phonological sensitivity relationship, the relationship between morphosyntax and phonological sensitivity has received less attention. Morphosyntax development is generally considered to be independent of phonological sensitivity development in children with NH, including in those children with NH who have weak language skills, to the extent that morphosyntax interventions have served as control conditions in assessment of phonological sensitivity training (e.g., Bus and van IJzendoorn, 1999; Bianco et al., 2010; Lyster et al., 2016). In our model of emergent literacy for children who use CIs, we believe these two constructs do maintain independence, as they do in children with NH. Again, much like vocabulary-phonological sensitivity, we suggest that both skills are influenced by auditory access and therefore their development in prereaders is likely to be correlated. We also note that the development of both skills in prereaders is related to future literacy success (Whitehurst and Lonigan, 1998; Hogan et al., 2005; Kirby et al., 2012; Lyster et al., 2016) and should therefore be targeted for intervention, but that improvements on morphosyntax will not lead to gains in phonological sensitivity.

### Co-development of Language Constructs

Oral language skills are a fundamental part of emergent literacy, supporting the development of other emergent literacy skills and future decoding abilities (Foorman et al., 2002). Given that the ultimate goal of emergent literacy is to progress from prereading to efficient decoding to successful reading comprehension, oral language has been shown to be predictive of reading comprehension (Catts, 1993; Lonigan and Shanahan, 2010; Mayberry et al., 2011). However, as summarized above, children who use CIs show significantly poorer performance on both vocabulary and morphosyntax. It is highly likely that these performance deficits can be attributed at least in part to children’s auditory access (Svirsky et al., 2000; Geers et al., 2009; Nittrouer et al., 2014), but we wish to briefly consider how vocabulary and morphosyntax might influence one another during development as this has implications for emergent literacy intervention paradigms for children who use CIs.

In children with NH, spoken language development is best captured by a two-factor model (Lonigan and Milburn, 2017). The two factors, vocabulary and morphosyntax, are mutually influential with growth in vocabulary corresponding to growth in morphosyntax and vice versa (McBride-Chang et al., 2005; Wagner et al., 2007; Ramirez et al., 2014). Not only do vocabulary and morphosyntax influence each other, but growth in both vocabulary and morphosyntax knowledge predicts future comprehension in school-aged readers (Quinn et al., 2015; Brimo et al., 2018).

Recent data suggest that vocabulary and morphosyntax are uncoupled in children with low vocabulary levels (Colé et al., 2018), where growth in vocabulary has no influence on growth in morphosyntax and vice versa. In addition to their documented delays in expressive morphosyntax, children who use CIs have lower vocabulary levels (Lund, 2016). Additionally, though auditory access is correlated to growth of both vocabulary and morphosyntax during preschool (Nicholas and Geers, 2018), there is nothing to indicate that growth in vocabulary, for example, influences growth in morphosyntax for this population. We therefore suggest that vocabulary and morphosyntax are potentially correlated in children who use CIs, but that development in these constructs is uncoupled. This would be a piece of the development that we have hypothesized for vocabulary and phonological sensitivity or morphosyntax and phonological sensitivity: the two constructs are not unrelated, but growth in one does not influence development of the other and the two are likely mutually influenced by the child’s auditory abilities.

When considering intervention, though vocabulary and morphosyntax may be uncoupled, both constructs are clearly important for supporting children’s decoding abilities (Apel and Lawrence, 2011). However, we do not believe that a generalized emphasis on spoken language performance is likely to lead to improvements in children’s vocabulary, morphosyntax, and decoding. Improving children’s access to sound, including early fitting and regular use of hearing devices, is one of the best predictors of receptive and expressive language outcomes (see Park et al., 2019 for CI users and Tomblin et al., 2015 for hearing aid users). The reality, however, is that many children continue to have reduced auditory access. For example, only half of the sample of CI users achieve full-time use (indexed as 80% of waking hours) by age 3 years, indicating that many children who use CIs are not receiving sufficient auditory access to drive emergent literacy development (Walker et al., 2019). Rather, children who use CIs are likely to require specialized intervention in both vocabulary and morphosyntax to promote academic success (Lonigan and Shanahan, 2010). This would include explicit practice on new word learning, morphology, and complex syntax (Moeller et al., 2007).

## Testing the Model Predictions

In the preceding section, we outlined our hypotheses for how emergent literacy factors might co-develop in preschoolers who use CIs compared to how they co-develop in children with NH. The critical difference between our predictions and other theoretical frameworks is the inclusion of a causal chain of development, where gains in one factor (e.g., alphabetic knowledge) have a direct impact on children’s gains on another factor (e.g., phonological sensitivity) (c.f., Lederberg et al., 2013). We argue a need to revise the current thinking about emergent literacy by including a causal chain of development. This is an important step toward effective intervention development, as it provides a framework in which early learned skills serve as a foundation for later-learned skills (Adamson et al., 2019). To this end, testing the hypotheses that we have laid out is crucial to refining our proposed model, clarifying potential emergent literacy mechanisms in preschoolers who use CIs and developing effective interventions to prepare children for literacy education.

Because we have hypothesized a causal chain of development any tests of our hypotheses must allow for inferences of causality. Many teams have recognized the usefulness of longitudinal data for tracking developmental progressions in children with hearing loss, including those who use CIs. Modeling longitudinal data using structural equation modeling would allow us to infer causal influences between emergent literacy constructs (for an example applying this approach to literacy data in children who use CIs see Connor and Zwolan, 2004). Presuming the modeling outcomes support our hypotheses, experimental approaches designed to test the interventions we have proposed would provide further confirmation of the causal chain.

## Conclusions

We have presented evidence demonstrating that children who use CIs show performance deficits on nearly every aspect of emergent literacy. Though this can be attributed in part to differences in children’s auditory experiences, the fact that many high schoolers who use CIs are not reading at grade level suggests that improving auditory skills alone will not be sufficient and interventions that specifically target literacy for children who use CIs are necessary. We argue that these interventions should emphasize emergent literacy in prereaders who use CIs, to reduce disparities in emergent literacy early, and give children who use CIs a foundation for literacy instruction. Here, we have laid out our framework to describe how the auditory access provided by the CI influences the co-development of emergent literacy factors. We have provided hypotheses on mechanism, as well as alternatives to several of those hypotheses. Within this framework, we have speculated on those interventions that may be most likely to produce emergent literacy gains if our hypotheses are supported. We recognize that many of these interventions remain to be explicitly tested and the long-term benefits of these interventions will need to be ascertained. Thus, though it is a cliché to wrap up this review with the statement, “More work is needed,” we look forward to empirical tests of our hypotheses and interventions, with the aim of improving literacy outcomes for children who use CIs.

## Author Contributions

All authors listed have made a substantial, direct and intellectual contribution to the work, and approved it for publication.

### Conflict of Interest

The authors declare that the research was conducted in the absence of any commercial or financial relationships that could be construed as a potential conflict of interest.
